# Queen's Jubilee Hospital

**Published:** 1905-02-25

**Authors:** R. P. Brooks

**Affiliations:** Late secretary of the medical committee of the Queen's Jubilee Hospital


### EDITOR'S LETTER-BOX.

[Our Correspondents are reminded that prolixity is a great bar to publication, and that brevity of style and conciseness of statement greatly facilitate early insertion.]

### QUEEN'S JUBILEE HOSPITAL.

*To the Editor of* The Hospital.

Dear Sir,—Would you kindly insert in your journal the following resolutions which were passed by the late members of the medical staff of the Queen's Jubilee Hospital on February 7th, 1905, and sent by them to the chairman of the general committee on the occasion of their withdrawal from the hospital staff.

Yours truly,
(Signed) R. P. Brooks.
Late secretary of the medical committee of the Queen's Jubilee Hospital.

The resolutions, which were carried unanimously, are as follows:—

(1) That the disorganisation in the nursing staff is due to the action of the board of management in retaining nurses that had been unfavourably reported on by the medical staff and the matron, and that no good matron could be retained under the circumstances.

(2) That we, the undersigned members of the medical staff, having watched the management of the hospital during some years think that there is no prospect of improvement under the present influences, and are therefore unable to continue our connection with the institution; but are willing to continue our attendance on the patients pending the appointment of successors within a reasonable time.

(Signed) A. M. Ware, M.D.
E. W. Miles, F.R.C.S.
J. Morrison, M.D.
Milsom Rees, F.R.C.S.
R. P. Brooks, F.R.C.S.
C. N. Barton, M.R.C.S., L.R.C.P.

